# Comparison of Traditional and Next-Generation Approaches for Uncovering Phytoplasma Diversity, with Discovery of New Groups, Subgroups and Potential Vectors

**DOI:** 10.3390/biology11070977

**Published:** 2022-06-28

**Authors:** Valeria Trivellone, Yanghui Cao, Christopher H. Dietrich

**Affiliations:** Illinois Natural History Survey, Prairie Research Institute, University of Illinois at Urbana Champaign, Champaign, IL 61820, USA; caoyh@illinois.edu (Y.C.); chdietri@illinois.edu (C.H.D.)

**Keywords:** anchored hybrid enrichment, biodiversity, biorepository, nested PCR, sanger sequencing

## Abstract

**Simple Summary:**

Phytoplasmas are bacteria transmitted by insects that cause severe diseases in many plants, including crops, worldwide. Most phytoplasma research focuses on the epidemiology of phytoplasma-associated diseases in agriculture, and relatively few efforts have been made to survey phytoplasma diversity in natural areas. We compared traditional methods for detecting and identifying phytoplasmas with a new method based on next-generation DNA sequencing and found that the next-generation method performs as well, or better, for identifying phytoplasmas in DNA extracted from plant-feeding insects. Using this method, we report several new country/region records and insect associations for known phytoplasmas, three new designated phytoplasma subgroups and three possible new groups.

**Abstract:**

Despite several decades’ effort to detect and identify phytoplasmas (Mollicutes) using PCR and Sanger sequencing focusing on diseased plants, knowledge of phytoplasma biodiversity and vector associations remains highly incomplete. To improve protocols for documenting phytoplasma diversity and ecology, we used DNA extracted from phloem-feeding insects and compared traditional Sanger sequencing with a next-generation sequencing method, Anchored Hybrid Enrichment (AHE) for detecting and characterizing phytoplasmas. Among 22 of 180 leafhopper samples that initially tested positive for phytoplasmas using qPCR, AHE yielded phytoplasma *16Sr* sequences for 20 (19 complete and 1 partial sequence) while Sanger sequencing yielded sequences for 16 (11 complete and 5 partial). AHE yielded phytoplasma sequences for an additional 7 samples (3 complete and 4 partial) that did not meet the qPCR threshold for phytoplasma positivity or yielded non-phytoplasma sequences using Sanger sequencing. This suggests that AHE is more efficient for obtaining phytoplasma sequences. Twenty-three samples with sufficient data were classified into eight *16Sr* subgroups (16SrI-B, I-F, I-AO, III-U, V-C, IX-J, XI-C, XXXVII-A), three new subgroups (designated as 16SrVI-L, XV-D, XI-G) and three possible new groups. Our results suggest that screening phloem-feeding insects using qPCR and AHE sequencing may be the most efficient method for discovering new phytoplasmas.

## 1. Introduction

Associations between hemipteran insects and phytoplasmas have evolved over the past ~350 million years [1] and vary from commensal to parasitic from the insect’s perspective. Most prior research on phytoplasmas has been conducted from the relatively narrow perspective of plant disease epidemiology in agro-ecosystems with a focus on the detection and characterization of phytoplasmas obtained from diseased plants. However, as has already been well documented for other groups of organisms, natural habitats worldwide continue to harbor large numbers of undiscovered taxa [2]. Recent discoveries of new phytoplasmas in natural areas worldwide suggest that the diversity and ecology of this ancient lineage of highly specialized bacteria remain poorly known [3,4]. Broader knowledge of phytoplasma diversity and phylogeny will be crucial to uncovering the evolutionary mechanisms that drive host shifts and evolution in this system, and for providing a framework to understand the potential of previously undiscovered phytoplasmas as future threats to agriculture [5]. Since bacterial cells preserved in fossil material, either inside their hosts or in natural resins (such as amber), are difficult to identify, phylogenetic and molecular divergence time analyses incorporating the extant diversity of both phytoplasmas and their insect hosts will be crucial for the reconstruction of the evolutionary history of host associations in this group.

Most of the phytoplasmas currently known have been discovered because they cause symptoms of diseases associated with phytoplasmas in cultivated plants. However, phytoplasma infections, particularly in natural areas, may be asymptomatic and therefore remain unnoticed [6,7]. Also, although phytoplasmas normally require an insect vector in order to spread from plant to plant, insect vectors remain unknown for most known phytoplasmas [8]. Thus, in addition to our poor overall knowledge of phytoplasma diversity in natural areas, there remain major gaps in knowledge of the ecology of known phytoplasmas. 

To accelerate the process of detecting and naming new phytoplasmas and documenting their associations with potential insect vectors, new, cost-effective approaches are needed. Recently, we used quantitative PCR (qPCR) to screen a large sample of phloem-feeding leafhoppers (227 species) collected in natural areas worldwide. We detected phytoplasmas in 3% of the samples and the phytoplasmas detected represented 6 previously unknown phytoplasma subgroups [3,4]. These results not only indicate that natural areas worldwide harbor a large diversity of undocumented phytoplasmas but also suggest that screening DNA extracted from phloem-feeding leafhoppers is an efficient method for discovering new phytoplasmas. In this paper, we explore the potential use of next-generation sequencing methods for detecting and characterizing phytoplasmas from leafhopper DNA samples and documenting new host associations.

Traditional methods for classification of phytoplasmas rely on the polymerase chain reaction (PCR) and Sanger sequencing of the *16S rRNA* (hereafter *16Sr*) gene and a few additional genes (e.g., *ribosomal protein, rp*) [9]. Next-generation sequencing methods have the potential to provide data from these genes as well as many other parts of the phytoplasma genome at low cost, providing additional discriminatory power for distinguishing strains as well as data useful for phylogenetics and comparative genomics. The anchored hybrid enrichment (AHE) approach [10] has been used successfully for phylogenomic analyses of a wide variety of organisms. This method uses hybridization probes to enrich DNA samples obtained from host organisms for specific regions of the genome, incorporating multiple probes per locus in order to capture variable gene regions useful for both coarse and finer-scale phylogenetic resolution and characterization [11,12]. The method is particularly useful for obtaining sequence data from organisms with large genomes that would be prohibitively expensive to sequence in their entirety. This method allows the loci of interest to be sequenced using next-generation DNA sequencing technology at a reduced cost compared to whole genome shotgun sequencing.

Given that bacteria have relatively small genomes compared to eukaryotes, entire bacterial genomes can often be sequenced at a low cost, assuming that pure cultures of the bacteria of interest can be obtained. Unfortunately, many bacterial endosymbionts, including phytoplasmas, cannot be cultured axenically and are, therefore, difficult to obtain as pure samples. Thus, genome sequencing for such bacteria has lagged far behind efforts to sequence free-living bacterial species. Target capture methods such as AHE provide a potential method not only for detecting and characterizing phytoplasmas in DNA samples obtained from potential host insects or plants but also for obtaining DNA from regions of the phytoplasma genome beyond the few gene regions used for traditional classification and identification of *Candidatus* species and *16Sr* phytoplasma groups and subgroups [13,14]. To our knowledge, the anchored hybrid method has not been used previously for phytoplasmas and we are aware of only one other use of this method for another group of endosymbiotic bacteria [15].

The aim of this study was to test the reliability of a new method coupling target capture with next-generation sequencing to detect and identify phytoplasmas in insect samples collected for biodiversity studies. We provide a new methodological protocol to analyze the diversity of phytoplasmas and also report the discovery of new strains. Our findings build on recent work suggesting that natural areas worldwide harbor a highly diverse assemblage of undocumented phytoplasmas [3]. 

## 2. Materials and Methods

### 2.1. Leafhopper Collection

For this study, we used genomic DNA samples, mostly ethanol-preserved museum specimens of leafhoppers (Hemiptera: Cicadellidae), from the Illinois Natural History Survey (INHS) insect collection previously analyzed for a project focusing on leafhopper phylogenetics [12,16]. 

The original samples included one or more individuals of a single species collected at the same locality and date, with collecting events occurring over the past 25 years and distributed in natural areas worldwide. Specimens were mostly collected directly into 95% ethanol in the field, then sorted according to species and stored in −20 °C freezers at the INHS prior to DNA extraction. Leafhopper specimens were identified as belonging to a specific species by examining diagnostic parts of the male genitalia and using published keys and related taxonomic literature.

### 2.2. Phytoplasma Detection and Identification

Total DNA was extracted non-destructively from each voucher specimen using a DNeasy Blood and Tissue Kit (Qiagen, Valencia, CA, USA) and following manufacturer protocols except for increasing the incubation time to 48 h. In most cases, only the abdomen was used for DNA extraction with the remainder of the body retained as a voucher. Extracted abdomens were subsequently placed in microvials with glycerin and stored with the dry-mounted voucher specimens. All voucher specimens are deposited in the Illinois Natural History Survey Insect Collection. The DNA templates were further processed with different approaches as described below.

#### 2.2.1. qPCR

In order to test the reliability of the pilot phytoplasma probes designed for AHE analysis (see Section 2.2.2), TaqMan real-time PCR (qPCR) analysis of the *16Sr* gene was carried out on DNA templates from 180 samples (1 sample per species), which represent additional biorepository specimens not screened by Trivellone et al. [3]. The 10 µL reaction volume contained 4 µL of DNA template diluted 1:2, 5 µL Platinum Quantitative PCR Supermix- UDG (Thermo Fisher Scientific, Waltham, MA, USA), 160 nM for each primer (Forward 5′-CGTACGCAAGTATGAAACTTAAAGGA-3′, Reverse 5′-TCTTCGAATTAAACAACATGATCCA-3′) and a probe (5′-TGACGGGACTCCGCACAAGCG-3′). The assays were performed in 96-well plates on a CFX96 thermal cycler (Bio- Rad, Hercules, CA, USA), according to the protocol of Angelini et al. [17]. DNA of the FD (Italian grapevine yellows) phytoplasma, provided by the Viticulture Research Centre (Conegliano, Italy), was used as a positive control in all amplification reactions. The quantification cycle (Cq) value was evaluated for each sample using the cut-off value reported by Trivellone et al. [3], i.e., samples with a Cq value ≤ 30.38 were tagged as ‘*phy*’ (positive detection for phytoplasma) and those with a Cq value > 30.38 were tagged as ‘*oBa*’ (positive detection of other bacterial DNA). All the samples which yielded a positive signal for the presence of bacterial *16Sr* gene were further analyzed using both the traditional approach, nested PCR followed by low-throughput Sanger sequencing and the new high-throughput next-generation approach (AHE).

#### 2.2.2. Anchored Hybrid Enrichment (AHE)

The performance of AHE results was assessed for samples testing positive for bacterial *16Sr* gene using qPCR.

Preparation of anchored hybrid libraries and paired-end 150 bp sequencing were performed at Rapid Genomics LLC (Gainesville, FL, USA) using the probe kit in Cao et al. [12], which contains ~50,000 probes mostly targeting insect genes but also a pilot set of 73 hybridization probes targeting *16Sr* gene of phytoplasmas. To maximize capture efficiency, probes were designed to incorporate genetic variation observed in phytoplasma *16Sr* sequence data available in NCBI. Raw reads were cleaned by TrimmomaticPE [18] to remove adaptors and poor-quality data with a minimum length of 50, leading and trailing settings of 5, sliding window setting of 4:15 and an Illuminaclip setting of 2:30:10. Then cleaned reads were assessed for quality using FASTQC [19]. Two methods were used to predict phytoplasma *16Sr*.

Method 1 (ABySS) performs de novo assembly first, then uses BLASTN searches against a reference phytoplasma *16Sr* sequence. This method was carried out in two steps. First, cleaned reads were assembled by ABySS v2.1.0 [20] with minimum mean k-mer coverage of three. Three assemblies were generated for each sample using k-mer length settings of 29, 50 and 90 bp, respectively (Step 1). Second, BLASTN searches were performed using a complete *16Sr* phytoplasma reference sequence (CP000061.1, [21]) against the anchored hybrid assemblies with a cut-off E-value of 10^−5^. Candidate sequences were then processed for BLASTN searches against all the bacterial *16Sr* sequences used for probe design, including *16Sr* genes of phytoplasma, three primary nutritional symbionts of leafhoppers (*Candidatus* Sulcia, *Candidatus* Nasuia, *Candidatus* Baumannia and *Candidatus* Cardinium), and four other leafhopper endosymbionts (*Wolbachia*, *Rickettsiella*, *Arsenophonus* and a *Sodalis*-like bacterium). Because this step generates a very large number of sequences, identification of which may be computationally intensive, especially for shorter sequences, an initial cut-off of 800 bp was applied for filtering, i.e., sequences with best-hit as phytoplasma *16Sr* and ≥800 bp were retained for further analysis (Step 2). For all the positive samples based on qPCR, candidate sequences matching phytoplasma *16Sr* in step 2 were processed in the further step (step 3, see below) regardless of their length.

Method 2 (HybPiper) is based on the HybPiper v2.0.1 pipeline [22] which first maps cleaned reads to reference sequences and then conducts de novo assembly using these reads. This method generally produces longer assemblies than Method 1; however, it is more suitable for protein-coding genes. Due to the fact that predicted sequences are automatically trimmed according to the reference sequence, this can result in false deletions within non-coding gene regions; thus the assemblies need to be checked manually when using this method to identify *16Sr*. We only employed Method 2 to detect phytoplasma *16Sr* in the positive samples based on qPCR.

First, cleaned reads were mapped to the phytoplasma *16Sr* reference sequence (CP000061.1) using the BWA method [23] in the HybPiper pipeline and then assembled by SPAdes [24] (Step 1). Second, assemblies were manually checked and corrected by viewing the reads mapping to the assemblies using IGV v2.12.3 [25] (Step 2).

For each method, the last step (Step 3) is to identify the candidate sequences through BLASTN searches against the standard nucleotide databases in GenBank. Sequences whose best hit was a phytoplasma *16Sr* were tagged as ‘*phy*’ (positive detection for phytoplasma); sequences whose hit was other bacteria were tagged as ‘oBa’. If no assembly was retrieved, the sample was tagged as ‘*neg*’ (negative for the presence of phytoplasma or other bacterial DNA). Phytoplasma *16Sr* genes retrieved by these two methods were aligned using MultiAlign software [26] to compare the sequence similarity.

#### 2.2.3. Nested PCR and Sanger DNA Sequencing

*16Sr* partial sequences were obtained by using nested PCR with universal primer pair P1A (5′-ACGCTGGCGGCGCGCCTAATAC-3′)/P7A (5′-CCTTCATCGGCTCTTAGTGC-3′) followed by three different primer pairs R16F2n (5′-GAAACGACTGCTAAGACTGG-3′)/R16R2 (5′-TGACGGGCGGTGTGTACAAACCCCG-3′), 16S-ycF1 (5′-CCTAATACATGCAAGTCGAACG-3′)/16S-ycR1 (5′-TTGGGGTTAAGTCGTAACAAGGTA-3′) and 16S-ycF3 (5′-TTTAGTGGCGAACGGGTGAGTAAC-3′)/16S-ycR3 (5′-GGTGGGGATGGATCACCTCCTTT-3′) (last two pairs designed in this study). The reaction mixture (10 μL for direct PCR and 20 μL for nested PCR) contained 1 μL of the diluted DNA template (1:5) or of the direct PCR product, 0.2 mM dNTPs, 0.6 μM each primer, 0.75 U PrimeSTAR GXL DNA polymerase (TaKaRa Bio, Kusatsu, Japan) and the buffer supplied with the enzyme. The following thermal cycling protocol was used for direct amplifications: initial denaturation at 98 °C for 1′, then 30 cycles of 30″ at 98 °C, 15″ at 55 °C and 2′ at 68 °C, and a final extension for 10′ at 72 °C. For nested PCR 35 cycles of 10″ at 98 °C, 15″ at 55 °C and 1:45′ at 68 °C, and a final extension for 10′ at 72 °C. Amplicons were visualized on 1% agarose gel under a GelDoc XR UV transilluminator (Biorad). The DNA of FD (Italian grapevine yellows) phytoplasma, provided by the Viticulture Research Centre (Conegliano, Italy), was used as a positive reference sample in all the amplification reactions. Products were submitted for Sanger sequencing to the Roy G. Carver Biotechnology Center of the University of Illinois. Forward and reverse reads were assembled using Gap4 and Pregap [27], followed by manual editing. The consensus sequences were evaluated using BLASTN and tagged as ‘*phy*’, ‘*oBa*’ or ‘*neg*’.

The sequences obtained from Sanger sequencing and the AHE sequence assemblies for the same samples were compared using MultiAlign software [26] and BLASTN to evaluate similarity, coverage and identity.

Final characterization and identification of the phytoplasma sequences obtained from both sequencing approaches were accomplished using ‘iPhyClassifier’ [28].

#### 2.2.4. Virtual RFLP Analysis of *16S rRNA* Genes

To identify phytoplasma strains, we used computer-simulated restriction fragment length polymorphism (RFLP) digestion of *16Sr* sequences and virtual gels as implemented in ‘iPhyClassifier’ (http://plantpathology.ba.ars.usda.gov/cgi-bin/resource/iphyclassifier.cgi (accessed on 5 June 2022)) [28]. The following modules in ‘iPhyClassifier’ were run: a pairwise comparison of each sequence with the reference strains of all previously described ‘*Ca*. Phytoplasma’ using BLAST, a *16Sr* group/subgroup classification based on restriction fragment length polymorphism (RLFP) analyses of 17 restriction enzymes included in the F2nR2 barcoding fragment of the *16Sr* gene, following the guidelines of the International Committee of Systematic Bacteriology Subcommittee for the Taxonomy of Mollicutes [29].

### 2.3. Phylogenetic Analysis

Nucleotide sequences used for phylogenetic analysis include 169 phytoplasma *16Sr* used by [1], which represent 35 groups and 169 designated subgroups, 23 phytoplasma *16Sr* sequences (≥1243 bp) newly predicted by the HybPiper method and the 1*6Sr* of *Acholeplasma brassicae* (FO681348.1, [30]) as an outgroup. Sequences were aligned using MAFFT [31] with an automatically selected alignment algorithm, followed by manual checking and editing. IQ-TREE v1.6.12 [32] was used for model selection with the -m TEST option and maximum likelihood analysis with 100 bootstrap replicates. TIM3 + F+I + G4 was determined as the best-fit model according to the Bayesian information criterion.

## 3. Results

Overall, 35 leafhopper specimens representing 35 species (9 undescribed) in 32 different genera (including 6 undescribed) of Deltocephalinae from 18 different Countries/Regions tested positive for the presence of bacterial *16Sr* gene during the initial screening using qPCR (Table S1).

### 3.1. Comparison of the Methods for the Detection and Sequencing of Phytoplasmas

To investigate the accuracy and sensitivity of the AHE method, we compared the results with those yielded by traditional methods for detection and identification of phytoplasmas, qPCR and nested PCR followed by Sanger sequencing.

#### 3.1.1. qPCR Results

Of the 180 specimens screened using qPCR, 35 (19.5%) yielded a signal for the presence of bacterial *16Sr* gene and 22 (12%) showed a Cq value ≤ 30.38 indicating the presence of phytoplasma (Table 1).

All 35 samples testing positive for bacterial *16Sr* were analyzed using both AHE and traditional PCR followed by Sanger sequencing.

#### 3.1.2. AHE Results

For 27 of the 35 samples, both AHE assembly methods yielded identical sequences (differing only in length), including all the sequences identified as phytoplasma *16Sr* (initial BLAST, Table S2). Sixteen of these samples yielded identical sequences of the same length and eleven yielded identical sequences, but method 2 yielded a longer sequence. Six samples (24FR, 28ZA, 30MX, 32BR, 33US and 34AU) yielded sequences from other bacteria from one or both methods. For sample 31PH, method 1 yielded an assembly less than 356 bp in length, and no assembly was obtained using method 2; and for 35BR no assembly was obtained using either method (Table S2). The sequences obtained with method 2 (Table 1) were considered for the final comparison with Sanger sequencing (see below Section 3.1.4) and included 23 nearly full-length *16Sr* phytoplasma sequences, 4 partial *16Sr* phytoplasma sequences, 4 sequences from other Bacteria and 4 negative samples. All AHE raw reads were deposited into the Sequence Read Archive (SRA) SRA database in NCBI (Accession numbers in Table S2).

#### 3.1.3. Nested PCR and Sanger Sequencing Results

Standard PCR was performed for the same 35 samples using the primer pair R16F2n/R16R2, but only 10 samples yielded amplicons of the expected size (1243 bp), with forward and reverse strands successfully sequenced for only three samples and the forward strand for one additional sample. For this reason, the new primer pairs 16SR16S-ycF1/16S-ycR1 and 16SR16S-ycF3/16S-ycR3 were designed and used to amplify the 35 same samples. The new primer pair 16SR16S-ycF1/16S-ycR1 yielded a total of 33 amplicons and for 22 sequences the expected size (1449 bp) was successfully obtained (both forward and reverse) and included all samples sequenced with R16F2n/R16R2. For one more sequence, the expected size (1443 bp) was successfully obtained with 16SR16S-ycF3/16S-ycR3 primer pair. Overall, the best BLAST hits included 11 phytoplasma sequences of the expected size, 5 phytoplasma sequences with only the forward or reverse strand (‘*phy-part*’, length ranged from 552 to 873 bp), and 19 sequences of other Bacteria (11 from both strands—‘*oBa*’—and 8 from the forward or reverse ‘*oBa*-part’) (Table 1). The fasta file including the sequences is available in the Illinois Data Bank (DOI: https://doi.org/10.13012/B2IDB-9804959_V1 (accessed on 27 June 2022)).

#### 3.1.4. Comparison among Methods of Detection and Sequencing

The comparison among the methods is summarized in Table 2, Tables S2 and S3. Based on the Cq value, the qPCR results are assigned to one of two categories: ‘*phy*’ or ‘*oBa*’. For AHE and nested PCR, the same categories are included with partial sequences indicated by ‘*phy-part*’ and ‘*oBa-part*’, respectively. Overall, by using the three methods for detection of phytoplasmas, 54% of the results were concordant, 43% samples were identified as phytoplasmas (15 samples) and 11% were identified as other bacteria (4 samples) (Table 2, gray cells). Among the 15 samples, 11 samples (2TH, 4CH, 5MX, 7IL, 8AU, 9TW, 10US, 13BR,17MN, 23CN and 27US) yielded the nearly full-length *16Sr* gene or the F2n/R2 *16Sr* fragment was obtained from both AHE and nested PCR. Pairwise comparison indicated that the sequences were identical or nearly so, with one to four bp differences and a maximum of two gaps. For three samples (1CH, 3AU, 6MG) the forward or reverse strand were obtained after sequencing of nested PCR templates and showed several bp differences compared with the AHE sequences. A single sequence (29CN), although blasted to phytoplasma in NCBI, yielded only a short fragment of *16Sr* for both AHE (620 bp) and Sanger sequencing (873 bp). For most samples, the *16Sr* sequence assembled from AHE data was longer than that obtained by nested PCR and Sanger sequencing (Table 1).

Among the four samples that were detected as other bacteria by all methods, BLAST yielded the following best hits: uncultured bacterium-HE577681.1 (sample 32BR), uncultured bacterium-KF071830.1 and *Staphylococcus hominis*-MZ014435.1 based on nested PCR and AHE sequences, respectively (sample 24FR), *Cutibacterium acnes*-AP022845.1 based on the AHE sequence only (sample 34AU), *Escherichia coli*-ON054387.1 based on a single strand of nested PCR and *Flavobacterium* sp.-MN960084.1 based on AHE sequence (30MX).

For a total of four samples, no sequences were obtained using AHE. Two of these tested positive for phytoplasma using qPCR but only one yielded a partial (552 bp) phytoplasma sequence while the other yielded a sequence from a non-phytoplasma bacterium. The other two did not meet the qPCR threshold for presence of phytoplasma and yielded non-phytoplasma bacterial sequences using Sanger sequencing. Among the other twelve samples that yielded inconclusive results because AHE yielded phytoplasma sequences but Sanger yielded non-phytoplasma bacterial sequences, for five samples (11FR, 12ZM, 15CA, 16FR and 26PH) AHE and qPCR yielded consistent results. For the remaining seven samples (14CN, 18MN, 19CD, 20PE, 21ZA, 22CH and 25AU) the qPCR and Sanger results were in agreement. Given that the AHE sequences of these twelve samples all matched a published phytoplasma *16Sr* in NCBI standard nucleotide databases with high identity (coverage ≥ 99% and similarity ≥ 98.5%), and most of these AHE sequences are complete (8 sequences) or nearly so (1 sequence > 1450 bp and 3 sequences 900–1235 bp), the likelihood that they resulted from an erroneous sequence assembly is extremely low. Thus, we are confident that these samples are phytoplasma positive even though Sanger sequencing yielded a conflicting, non-phytoplasma sequence. To test whether the Sanger sequence could also be recovered from these samples by the AHE methods, i.e., indicating that both phytoplasma and additional bacteria (as detected by Sanger sequencing) are present in the same sample, the non-phytoplasma Sanger sequences were used as a reference to screen for highly similar sequences in the AHE data (same two methods used to screen for phytoplasma *16Sr* but with a different reference). Two samples yielded AHE assemblies (a 640 bp assembly for 12ZM and a 901 bp assembly for 25AU) identical or nearly so to the Sanger sequences, indicating that both phytoplasma and another bacterium are present in these samples. We failed to retrieve sequences highly similar to the Sanger sequences in the other 10 samples, possibly because the *16Sr* sequences of these bacteria are too divergent to have been captured by the specific AHE probes included in our study. Although mixed phytoplasma infections have sometimes been reported for single individual insects, we found no clear evidence for mixed phytoplasma infections in any of the samples tested. The high stringency of the AHE assembly methods used would have yielded separate assemblies for multiple phytoplasmas if they had been present in the samples. Moreover, in all the cases where the Sanger method yielded a consensus sequence there was no evidence for multiple bacterial DNA strains.

Using qPCR, AHE and nested PCR followed by Sanger sequencing, the 14 congruent phytoplasma nearly full-length *16Sr* sequences and 9 sequences obtained by AHE only were further classified using ‘iPhyClassifier’ (see below).

### 3.2. Characterization and Identification of Phytoplasma Strains

The 14 AHE sequences that yielded a consensus (i.e., were identical or nearly so) among all three methods and cover the nearly full-length *16Sr* gene were submitted to the first ‘iPhyClassifier’ database for ‘*Candidatus* (*Ca*.) Phytoplasma (P.)’ species assignment and then to the second database for group and subgroup classification. Six sequences (4CH, 5MX, 13BR, 17MN, 23CN and 27US) were most closely related to the reference strain ‘*Ca*. P. asteris’ (GenBank accession: M30790) sharing 99.4%, 99.5%, 99.8%, 99.8%, 99.9% and 99.7% sequence similarity respectively. These six phytoplasma strains belong to the aster yellows (16SrI) group and were further classified in two phytoplasma subgroups 16SrI-F (sample 4CH-*Euscelidius variegatus*) and 16SrI-B (13BR-*Dalbulus maidis*, 17MN-*Macrosteles guttatus,* 23CN-*Amimenus mojiensis* and 27US-*Graminella sonora*) all sharing 100% similarity. The sequence from 5MX (*Scaphytopius aequus*) has been assigned as a variant of 16SrI-B sharing the 98% similarity (Table 3). One sequence is closely related to ‘*Ca*. P. cynodontis‘ (10US-*Diplocolenus evansi*, 97.9%) and belonged to 16SrXI-C (100% similarity). The sequence from 3AU- *Orosius argentatus* showed 97.3% similarity with ‘*Ca*. P. trifolii’ and the subgroup assignment showed that it belonged to 16SrXXXVII-A (100% similarity with the reference strain AJ289192). Three sequences are closely related to ‘*Ca*. P. trifolii’ (1CH, 99.4% similarity), ‘*Ca*. P. phoenicium’ (7IL, 98.9%) and ‘*Ca*. P. brasiliense’ (8AU, 97.6%), and were classified in the group 16SrVI, 16SrIX and 16SrXV. The sample 1CH (*Rhopalopyx elongate*) was closely related to 16SrVI-D (96% similarity), the sample 7IL (*Neoaliturus argillaceus*) is similar to 16SrIX-J (97%), and the sample 8AU (*Micrelloides* n. sp.) is closely related to 16SrXV-C (89%); these three may represent new subgroups. Three sequences are similar to ‘*Ca*. P. trifolii’ (2TH, 97.2%) and Sorghum bunchy shoot phytoplasma (6MG, 96,5% and 9TW, 96,4%) and all may represent new groups. Regarding the subgroup assignment, the sample 2TH is 77% similar to 16SrXXXII-D, 6MG is 80% similar to 16SrXXIV-A and 9TW is 81% similar to 16SRXXIV-A.

An additional five AHE sequences agreed with qPCR for positive phytoplasma detection, among them two sequences (11FR- *Synophropsis lauri* and 12ZM-*Abimwa* sp.) were closely related to ‘*Ca*. P. pruni’ (*rrn*A) (reference strain JQ044393) with 99.1% and 99% similarity and represent a variant of the 16SrIII-U subgroup with 98% similarity.

One sequence (15CA- *Limotettix urnura*) is closely related to ‘*Ca*. P. cirsii’ (JQ044393) with 98.9% similarity and the 16SrXI-E subgroup is the closest related strain with a 93% similarity and may represent a new subgroup. The sequence 16FR (*Doratura homophyla*) was closely related to ‘*Ca*. P. asteris’ with 99.7% similarity and belonging to 16SrI-B with 100% similarity. The sequence 26 PH (from a not yet described leafhopper genus) shares 94.9% similarity with ‘*Ca*. P. asteris’ and was assigned to 16SrI-AO with 100% similarity. 

Seven sequences were detected as phytoplasma by AHE only, suggesting that this method may, in some cases, be more sensitive than traditional Sanger sequencing. Among these, three were not further characterized because they did not include the entire F2n/R2 fragment of *16Sr* required by ‘iPhyClassifier’ (Table 1). Among the remaining four sequences, three of them (14CN-*Nakaharanus bimaculatus*, 20PE-*Exitianus obscurinervis*, 21ZA-*Aconurella prolixa*) are closely related to ‘*Ca*. P. pruni’ (*rrn*A) with 99% similarity and representing a variant of the 16SrIII-U subgroup with 98% similarity. The last sequence (22-CH-*Osbornellus auronitens*) has 99.9% with the reference strain of ‘*Ca*. P. vitis’ (an incidental citation) and belongs to the 16SrV-C subgroup (100% similarity).

**Figure 1:**
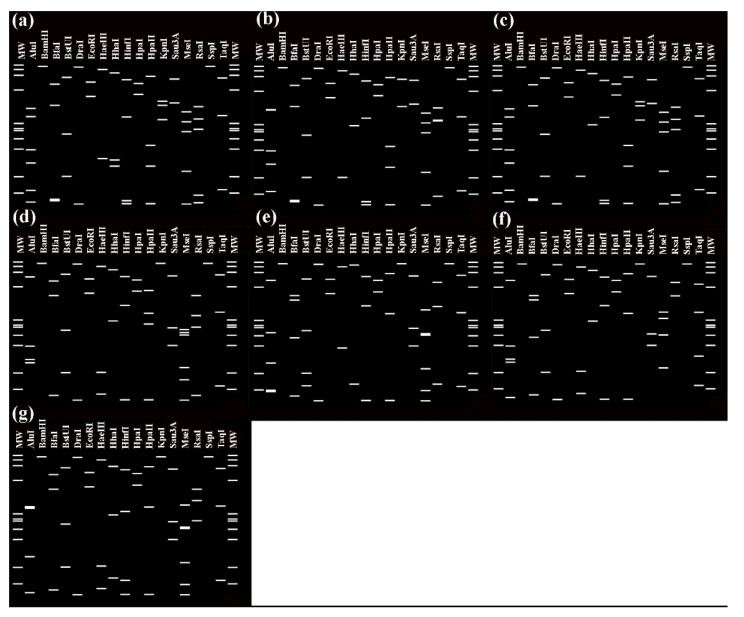
Distinct virtual RFLP patterns from in silico digestion of 16Sr gene F2nR2 fragments of the eight sequences belonging to group 16SrI detected in this study. (**a**) Samples 5MX, 13BR, 16FR, 17 MN, 23CN and 27US RFLP pattern of 16SrI-B, (**b**) Sample 4CH, 16SrI-F, (**c**) Sample 26PH, 16SrI-AO, (**d**) Samples 11FR, 12ZM, 14CN, 20PE, 21ZA, a variant of 16SrIII-U, (**e**) Sample 22CH, 16SrV-C, (**f**) Sample 10US, 16SrXI-C, (**g**) Sample 3AU, 16SrXXXVII-A. Recognition sites for the following 17 restriction enzymes were used in the simulated digestions: *AluI*, *BamHI*, *BfaI*, *BstUI* (*ThaI*), *DraI*, *EcoRI*, *HaeIII*, *HhaI*, *HinfI*, *HpaI, HpaII*, *KpnI, Sau3AI* (*cMboI*), *MseI*, *RsaI*, *SspI*, and *TaqI*. MW, φX174 DNA-*HaeIII* digestion as a marker.

The virtual RFLP pattern derived from the query *16Sr* gene R16F2n/R16R2 fragment for the four the sequences classified in group 16SrI are shown in Figure 1. 

**Figure 2:**
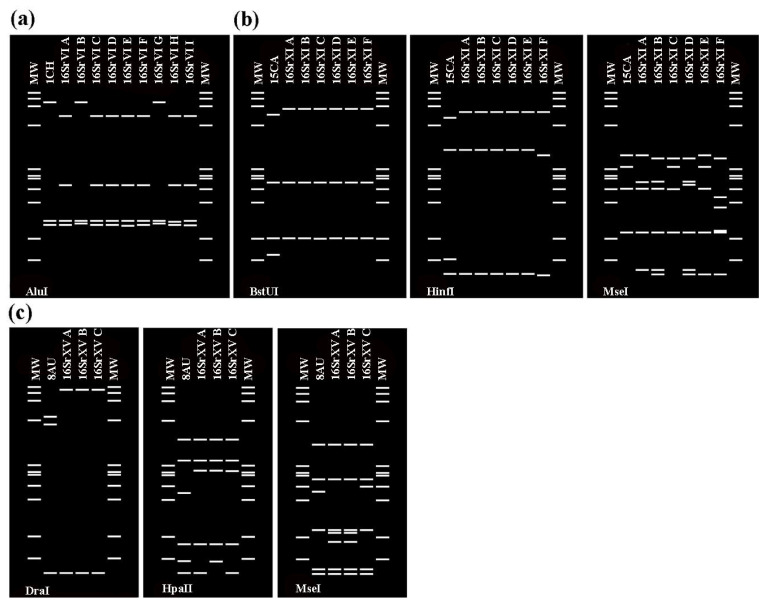
Identification of three new subgroups belonging to three different groups (16SrVI, 16SrXI, 16SrXV) based on the in silico RFLP patterns derived from key restriction endonucleases. (**a**) The new subgroup 16SrVI-L from the sample 1CH can be differentiated from the other nine subgroup profiles of the 16SrVI group by *AluI* restriction endonuclease. (**b**) The new subgroup 16SrXI-G from the sample 15CA can be differentiated from the other six subgroup profiles of the 16SrXI group by *BstUI, Hinf* and *MseI* restriction endonucleases. (**c**) The new subgroup 16SrXV-D from the sample 8AU can be differentiated from the other three subgroup profiles of the 16SrXV group by *DraI, HpaII* and *MseI* restriction endonucleases. MW, φX174 DNA-*HaeIII* digestion as a marker.

Three samples belonging to 16SrVI, XI, and XV exhibited a collective RFLP profile different from those of all previously established subgroups in each group (Figure 2). Sample 1CH (*Rhopalopyx elongata*) showed a different RFLP profile with restriction endonuclease *AluI* and was designated as a new subgroup 16SrVI-L (Figure 2a). Virtual RFLP profiles from *BstUI*, *HinfI* and *Mei* digestions distinguish the strain 15CA (*Limotettix urnura*) from all three previously designated 16SrXI subgroups and was designated as a new subgroup 16SrXI-G (Figure 2b). Similarly, profiles from *DraI*, *HpaII* and *MseI* digestions separate the strain from sample 8AU (*Micrelloides* n. sp.) and was designated as a new subgroup 16SrXV-D (Figure 2c). Sample 7IL (*Neoaliturus argillaceus*) returned a similarity coefficient of 97%, which is the threshold point for delineation of a new subgroup RFLP pattern type within a given group but is not distinct based on any of the 17 restriction endonucleases It has been assigned to the 16SrIX-J subgroup.

**Figure 3:**
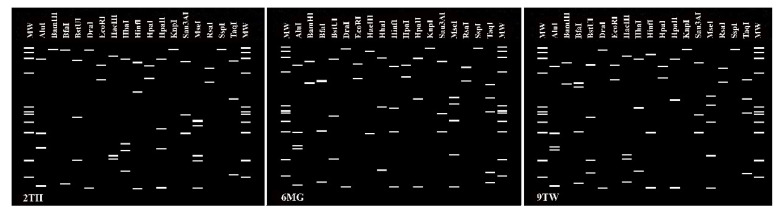
Distinct virtual RFLP patterns from in silico digestion of *16Sr* gene F2nR2 fragments of the 3 sequences that may represent new *16Sr* phytoplasma groups. From left, samples 2TH, 6MG, 9TW. Recognition sites for the following 17 restriction enzymes were used in the simulated digestions: *AluI*, *BamHI*, *BfaI*, *BstUI* (*ThaI*), *DraI*, *EcoRI*, *HaeIII*, *HhaI*, *HinfI*, *HpaI, HpaII*, *KpnI, Sau3AI* (*MboI), MseI*, *RsaI*, *SspI* and *TaqI*. MW, φX174 DNA-*HaeIII* digestion as a marker.

The virtual RFLP profiles of the 16S rDNA F2nR2 fragment of the phytoplasma strain from samples 2TH, 6MG and 9TW (Figure 3) have similarity coefficients of 0.77, 0.80 and 0.81 or less to the profiles of all previously recognized *16Sr* groups. Nevertheless, even if the first criterion for defining a new group is fulfilled (i.e., similarity coefficient is below the demarcation threshold of 0.85), we are not yet able to designate these strains as new groups because we cannot yet satisfy the second criterion to propose a new group [33]; that is, the new group must include at least one named species of the provisional genus ‘Ca. Phytoplasma’.

**Figure 4:**
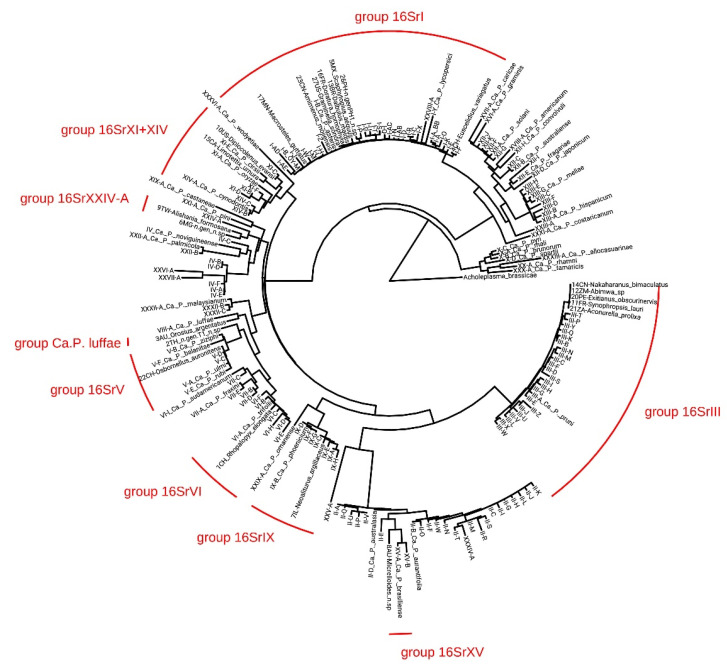
Maximum-likelihood tree of the *16S rRNA* gene for 169 phytoplasma strains from GenBank, 23 phytoplasma *16Sr* sequences (≥1243 bp) newly predicted by the HybPiper method and *Acholeplasma brassicae* (FO681348.1) (outgroup). Clades containing the 23 samples from this study are indicated in red.

The phylogenetic relationships of the 23 AHE phytoplasma sequences identified using ‘iPhyClassifier’ (Table 3), two samples whose Sanger sequences blasted to non phytoplasma bacteria (Table 1), and the reference strains for known phytoplasma groups/subgroups/species are shown Figure 4.

Sequences from the *16Sr* obtained in this study clustered in eight monophyletic phytoplasma clades. The 16SrI group clade includes eight sequences from this study identified as 16SrI-B (5MX, 13BR, 16FR, 17MN, 23CN and 27US) which grouped with the cluster including ‘*Ca*. P. asteris’ (M30790.1), one sequence identified as 16SrI-F (4CH) grouped with the reference strain of Apricot chlorotic leaf roll phytoplasma (AY265211.1), and another (26PH) identified as 16SRI-AO was identical to the reference strain of this recently described subgroup [4] and grouped with a phytoplasma strain isolated in Croatia (AF503568.1). The 16SrXI phytoplasma group clade includes two sequences from this study: the first identified as 16SrXI-C (10US) grouped with the strain detected in Germany (X76429.1); the second sequence designated as subgroup 16SrXI-G (15CA) grouped with a strain isolated in the Czech Republic (KR869146.1). Two more sequences (6MG and 9TW) which may represent new phytoplasma groups grouped with Sorghum bunchy shoot phytoplasma strain (AF509322.1). Sample 3AU, identified as 16SrXXXVII-A, was grouped with Loofah witches’-broom phytoplasma strain (AF086621.2) from Taiwan. The 16SrV phytoplasma group clade includes three sequences from this study: the first (22CH) identified as 16SrV-C was grouped with the reference strains of Elm yellows phytoplasmas (AY197655.1), Flavescence dorée (AY197645.1, AJ548787.2) and Rubus stunt phytoplasma (AY197648.1); the same clade also includes the two sequences (18MN and 19CD) that did not yielded congruent results between the two sequencing approaches and qPCR. The 16SrVI phytoplasma group clade includes 1CH from this study, designated as the new subgroup 16SrVI-L. The 16SrIX phytoplasma group clade includes 7IL from this study identified as 16SrIX-J, which is closely related to Pigeon pea witches’-broom (AF248957.1) subclade, ‘Juniperus occidentalis’ witches’-broom (GQ925918.1) and Brazilian Huanglongbing phytoplasmas (EU266074.1). The 16SrXV phytoplasma group clade includes 8AU from this study designated as new subgroup 16SrXV-D and is closely related to the subclade of Hibiscus witches’-broom (AF147708.1) and ‘Guazuma ulmifolia’ witches’-broom phytoplasmas (HQ258882.1). The 16SrIII phytoplasma group clade includes five sequences from this study identified as a variant of 16SrIII-U (11FR, 12ZM, 14CN, 20PE and 21ZA) which grouped together in the same subclade.

### 3.3. Insect-Phytoplasma Associations

**Figure 5:**
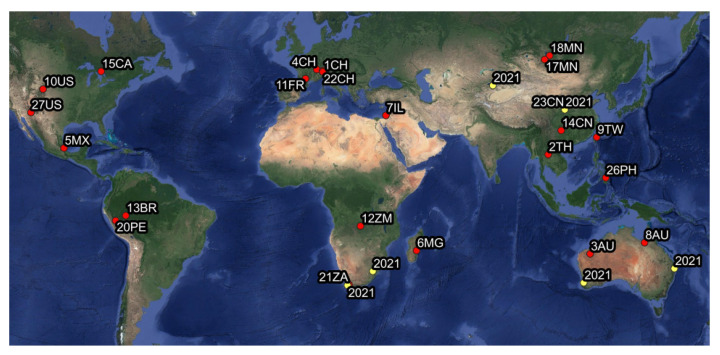
Map of the sampling sites of the 41 leafhopper specimens found positive for the presence of phytoplasmas, 35 individuals from the present study (points in red) and 6 individuals from the previous study [3] (points in yellow, 2021) (Trivellone et al., 2021). Map created QGIS 3.8 and was modified with Adobe Photoshop CC 2019. This map is licensed under an X/MIT style Open Source License by the Open Source Geospatial Foundation. For the acronyms see caption in Table 1.

Considering the results from the previous study [3], this study further increases the number of leafhopper specimens from a biorepository that tested positive for the presence of phytoplasma to a total of 37 (10 in 2021 and 27 in the present study). In this study the percentage of specimens testing positive after the initial screening using qPCR was 12%, four times higher than the percentage recovered using qPCR screening in previous study [3]. Moreover, three new subgroups and three tentatively new groups were discovered. The samples analyzed in this study were all collected in natural or semi-natural areas with low anthropogenic pressure. The 27 leafhopper specimens that tested positive in the present study (Table 1) were collected in 17 countries/regions: Australia, Brazil, Canada, Switzerland, China, France, Israel, Madagascar, Mongolia, Mexico, Peru, Philippines, Thailand, Taiwan, United States, South Africa and Zambia (Figure 5). This further illustrates the high diversity of previously unknown phytoplasmas in natural areas worldwide.

In this study, phytoplasmas in the aster yellows group (16SrI) were found in six different leafhopper species. The association between *Dalbulus maidis* (from Brazil) and aster yellows phytoplasma was already known in America and epidemiological studies to prevent spreading of aster yellows in Brazil have already been conducted [34]. The specimen that tested positive in our study was collected in a rainforest area. *Macrosteles guttatus* (from Mongolia) has never been tested for the presence of phytoplasmas; however, several other species this large, cosmopolitan genus is well-known for its association with aster yellows phytoplasmas [35]. Our sample was collected in a riparian sedge meadow. *Scaphytopius aequus* (from Mexico) is a species endemic to Mexico and the association of this species with aster yellows phytoplasma is reported here for the first time, although a few other species of *Scaphytopius* are known as competent vectors of phytoplasmas belonging to groups 16SrI and III. This species was collected in a secondary, montane rainforest. *Graminella sonora* (from USA, Arizona), a widespread grass-feeding species, tested positive for phytoplasma here for the first time; its congener, *Graminella nigrifrons* is a known vector of aster yellows phytoplasmas in the USA [36]. *Doratura homophyla* (from France), a grass-feeding species, is recorded in association with phytoplasmas for the first time and was collected on herbaceous vegetation in a location entirely surrounded by crop fields (e.g., vineyards). *Amimenus mojiensis* (from China), a genus and species not previously known as a phytoplasma host was collected on the same location and during the same collecting event (Figure 5) as *Acharis ussuriensis*, a species previously found to be infected by a new phytoplasma subgroup 16SrXIV-E [3,4]. *Euscelidius variegatus* (from Switzerland) was found associated with 16SrI-F and was collected on the interrow herbaceous layer of a vineyard. This is the first time this phytoplasma strain has been recorded for Switzerland; however, the same association was already recorded in the Czech Republic [37]. A new genus in the tribe Scaphoideini collected in the Philippines in a preserved native forest was found associated with the recently designated subgroup 16SrI-AO [4] that was detected in Kyrgyzstan in *Macrosteles sordidipennis* [3].

A total of five species were found to be infected with a variant of 16SrIII-U phytoplasma, all representing new associations*. Synophropsis lauri* (form France) was collected in the same agricultural location where *Doratura homophila* was collected. In Africa, two species were found to be infected by 16SrIII-U variant: *Abimwa* sp., which was collected in a preserved national forest, and *Aconerella prolixa* in an agricultural area. To our knowledge, only one 16SrIII group strain published on NCBI (AF056095) has been previously reported from Africa. *Nakaharanus bimaculatus* was collected in a natural protected area and this species is only known from China.

One species in the tribe Scaphoideini, *Osbornellus auronitens*, was found to be infected with a strain closely related to 16SrV-C. The association of other Scaphoideini with group 16SrV, and in particular -C and -D Flavescence dorée phytoplasma strains, is well-known in Europe, e.g., the Nearctic species *Scaphoideus titanus*. Interestingly, *Osbornellus auronitens* is also a Nearctic species recently recorded in Europe [38] and this specimen was collected by the first author in a sample representing the first European record in a woody patch surrounded by vineyards. The vineyard close to the patch is heavily affected by Flavescence dorée phytoplasma 16SrV-D, and other exotic species were found harboring both strains 16SrV-C and -D [39,40,41]. However, it is not possible to speculate further on these results because specific strains of Flavescence dorée phytoplasmas cannot be distinguished based on the 16S gene alone.

*Orosius argentatus* is a well-known vector of phytoplasmas in Australia [42] and in this study a specimen collected in a natural area was found to be infected with a strain identical to the reference strain of a recently described *Candidatus* species ‘*Ca*. P. stylosanthis’ [43]. 

*Diplocolenus evansi* was found infected with the 16SrXI-C phytoplasma and was collected in a riparian forest in Colorado (USA). This is the first record of phytoplasma infection in this leafhopper genus. Another specimen, *Limotettix urnura*, collected from a wetland in the Great Lakes region of Canada, was found infected with a strain designated here as 16SrXI-G in Canada and collected in a wetland. To our knowledge, this is the first record of a phytoplasma in this group in the Nearctic region.

A phytoplasma belonging to group 16SrXV was first reported in Australia in our previous study [3] and was designated as a new subgroup 16SrXV-C [4] associated with the leafhopper *Mayawa capitata* collected in Southwest Australia. For this phytoplasma group, we provide here a second record of the newly designated phytoplasma subgroup 16SrXV-D associated with another grass-feeding genus, *Micrelloides* (a new undescribed species) collected in northeast Australia in a riparian forest. This is also the first record of an association of this leafhopper genus with phytoplasmas. 

*Rhopalopyx elongata* was found to be associated with the new designated subgroup 16SrVI-L and was collected in a natural protected wetland in Switzerland [44]. To our knowledge, this is the first record of this association and this phytoplasma group is reported here newly recorded from Switzerland. 

*Neoaliturus argillaceus* was found to be infected with a strain closely related to 16SrIX-J in Israel. The association of closely related species in the genus *Neoaliturus* and phytoplasmas in group 16SrIX were recorded earlier in studies discussing the role of these species in transmitting phytoplasma diseases in carrots [45] and ornamental plants [46]. In this study the specimens were collected on desert vegetation.

## 4. Discussion

### 4.1. AHE Next Generation Sequencing as a Reliable Method for Future Phytoplasma Studies

Using AHE we obtained 23 well assembled *16Sr* sequences from 35 samples that tested positive for phytoplasma in DNA extracted from the bodies of sap-sucking hemipteran insects. To our knowledge, this is the first time the barcode region of the *16Sr* gene for phytoplasmas has been assembled using AHE sequencing, enabling identification of the phytoplasmas present. For the samples included in our study, AHE generally outperformed traditional nested PCR and Sanger sequencing, yielding *16Sr* contigs of greater length for most samples and indicating 100% (or nearly so) of sequence identity between contigs obtained from different sequencing methods. Interestingly, AHE yielded phytoplasma sequence data even in some cases (seven samples) where the sample failed to meet the required qPCR threshold for positive detection of phytoplasma. All but one of these showed a Cq close to the used threshold and not exceeding 33, indicating that it could be considered for eventual further characterization. However, these samples yielded sequences from other bacteria using traditional Sanger sequencing. These results may be due to several factors such as the concentration of phytoplasma in the sample relative to other bacteria, and the particular *16Sr* phytoplasma group present. In fact, even if the primers used, both in direct and nested PCR, target hypervariable regions of *16Sr* upon which the classification of bacteria relies, it was previously shown that the highest specificity and sensitivity does not exceed about 54% and 76%, respectively [47] and that each hypervariable region has different discriminatory power for different taxa [48]. Evidence for low resolution of the *16Sr* gene in bacterial classification, particularly for species-level discrimination, has been reported for several bacteria, e.g., *Bacillus* [49] and for certain groups of phytoplasmas, e.g., 16SrV within which subgroups share 98.6–99.9 % similarity [9]. The sometimes-conflicting results between AHE and Sanger sequencing highlights possible advantages of the AHE approach in cases where multiple bacterial species may be present in the same sample. In most cases where the results from the two methods conflicted, the sequence obtained from AHE was identified (via BLASTN) as a phytoplasma while the one obtained from Sanger sequencing was identified as a non-phytoplasma bacterium. This conflict may have resulted because the hybridization probes targeting phytoplasmas have higher specificity to phytoplasmas (due to greater length of each probe) and incorporate more sequence variation present across known phytoplasmas (due to the large number (>100) of probes used) than the PCR primers used for Sanger sequencing. PCR primers are relatively short (usually <25 bp) and target only highly conservative regions of the gene of interest. Thus, PCR-based methods may be more likely than AHE to yield sequences from non-target bacteria due to the difficulty of designing primers that specifically target phytoplasmas but exclude other kinds of bacteria. This suggests that next-generation sequencing methods may be more effective at obtaining data for phytoplasmas from DNA extracted from host insects than traditional PCR-based methods.

The anchored hybrid probe kit used in our study was designed primarily to obtain data for various regions of the host insect genome thought to be informative of phylogenetic relationships among these insects. Relatively few bacterial probes were included and these targeted only the *16Sr* and a few other bacterial genes. Nevertheless, our success at obtaining high-quality phytoplasma *16Sr* sequence data using this method matches the data obtained from the same samples using traditional PCR-based sequencing methods, indicating that the AHE approach is feasible for obtaining data from this gene as well as other parts of the phytoplasma genome. We are currently investigating the use of this method to obtain data from several other phytoplasma genes, as well as data on potential host plants [16] using samples of DNA extracted from potential host insects. We anticipate that this method will facilitate further refinements to current protocols for identifying and characterizing phytoplasmas as well as improve knowledge of phytoplasma evolution by providing more robust, multi-locus phylogenetic estimates. As pointed out by other authors the exclusive use of *16Sr* sequence data for *Candidatus* species or *16Sr* group/subgroup classification has limitations related to the inability of this gene to distinguish closely related strains and conflicting typing results due to the presence of two copies of the *16S rRNA*-encoding gene [50,51]. 

### 4.2. Uncovering New Phytoplasma Strains and Host Associations

Using the ‘iPhyClassifier’ database we designated three new phytoplasma subgroups: 16SrVI-L, 16SrXI-G and 16SrXV-D. Three new strains that may represent new phytoplasma groups were also reported here. Another four potentially new phytoplasmas strains could not be fully characterized because we were not able to obtain the entire F2nR2 sequence fragment required. We also reported three new groups of phytoplasmas that require further investigations in order to be properly classified. These results increase the number of new strains of phytoplasmas discovered from leafhoppers collected in natural areas. Most previously known phytoplasmas have been discovered by screening plants exhibiting symptoms thought to be typical of phytoplasma disease in agro-ecosystems. The new associations with phytoplasmas in subgroup 16SrI-B, aster yellows phytoplasma, confirm the ubiquitous distribution of this strain and further expand the list of potential vectors. Indeed, two leafhopper species that tested positive for 16SrI-B belong to the tribe Macrostelini which includes other species that are known to be vectors of aster yellows phytoplasmas. Although these species are restricted to different biogeographic areas, all of them have in common the same relationship with 16SrI-B suggesting that some traits conferring vector competence were acquired by the common ancestor of the tribe [52]. However, repeated detection of 16SrI-B phytoplasmas in species of other leafhopper tribes suggest that this strain has a high potential for expanding its niche through ecological fitting [53].

The phytoplasmas of group 16SrIII are known to be associated to X-disease of stone fruits affecting economically important orchards [54]. However, subgroup 16SrIII-U has mainly been found to be associated with herbaceous plants, including solanaceous crops, and was thought to be restricted to South America. However, Pérez-López et al. [55] recently reported variants of this subgroup from North America and also suggested a broader distribution. Our results further support this hypothesis with new detection in other biogeographic regions. We also uncovered associations with new potential vectors representing four distantly related leafhopper tribes: Athysanini, Fieberellini, Chiasmini and Selenocephalini. Species from the last two tribes have never been reported to be infected with phytoplasmas in group 16SrIII. ‘*Ca*. P. stylosanthis’ was recently described [43] and restricted to Australia where it is associated with diseases on *Stylosanthes scabra*, an economically important plant used for improvement of native pastures [56], *Carica papaya* and *Solanum tuberosum*. The association with *Orosius argentatus* was expected because stylo is a known host plant [57] and this leafhopper is also a known vector of phytoplasmas in group 16srII which are found in mixed infections on the same host plants [58].

Current knowledge of phytoplasma diversity is, therefore, likely to be highly biased by a prior emphasis on anthropogenic habitats with relatively few efforts having been undertaken to survey for phytoplasmas in natural areas. Since phytoplasmas have been co-evolving with their host plants and insect vectors for hundreds of millions of years [1], we predicted that phytoplasma diversity should be high in natural ecosystems worldwide. As we have shown, phloem-feeding insects collected in natural areas worldwide do, in fact, harbor many previously unknown phytoplasma strains. Such associations have remained undetected because most phytoplasma research continues to focus on plant disease epidemiology in agricultural systems and many phytoplasma infections in natural areas are asymptomatic.

Due to the fact that phytoplasmas are phloem limited and phloem-feeding hemipterans, including leafhoppers and psyllids, feed directly on phloem sap, these insects regularly acquire phytoplasmas by feeding on infected plants. During feeding, phytoplasma titer may increase in the insect’s body, and for competent vectors the titer is four times higher in the head than in the rest of the body [59,60], making detection easier. Thus, screening specimens of phloem-feeding insects belonging to groups known to include phytoplasma hosts and vectors may provide an efficient and productive way to detect phytoplasmas in various habitats. Previous studies that used standard molecular methods to screen for the presence of phytoplasmas in insects mostly focused on a few species in agroecosystems with ongoing phytoplasma disease outbreaks. These studies have reported the presence of phytoplasmas in 0–52% of the individual insects tested (e.g., 33–37.5% [61], 7–13% [62] 0–52% [63], 0–35% [64], 6–50% [65]). However, such studies usually employ extensive resources and sampling over a prolonged timespan during the growing season and target a single strain of phytoplasmas and a single or few species of host plants (crops). In such situations, phytoplasmas are concentrated in a specific habitat and host, yielding much higher detection rates than would be expected by a random screening of insects or plants in natural ecosystems. The relatively high rates of phytoplasma detection revealed by our screening of DNA extracted from phloem-feeding insects suggest that our approach offers the most efficient and cost-effective means for discovering and characterizing new phytoplasmas. The proportion of insect samples yielding phytoplasma sequences in the present study (12%) is even higher than that of our previous study [3,4].

## 5. Conclusions

Our study provides further evidence that phloem-feeding insects inhabiting natural areas worldwide harbor a diverse and largely undocumented diversity of phytoplasmas. We also demonstrate that the anchored hybrid enrichment (AHE) approach, incorporating next-generation DNA sequencing, outperforms traditional nested PCR and Sanger sequencing for detecting and characterizing phytoplasmas in samples of DNA extracted from potential insect vectors. So far, our screening efforts have focused on a single leafhopper subfamily, Deltocephalinae, which includes most of the known phytoplasma vectors. Given the availability of a large collection of ethanol-preserved Auchenorrhyncha at INHS, we also plan to screen representatives of additional cicadellid subfamilies as well as other groups of phloem-feeding hemipterans using the methods described above. We anticipate that these efforts will greatly expand our knowledge of phytoplasma diversity and host associations.

## Figures and Tables

**Table 1:** List of samples analyzed for the presence of phytoplasmas using AHE, qPCR and nested PCR followed by Sanger sequencing (nPCR-SS) methods. Description of locations and collecting events are in Table S1. The Code is an integer followed by the ISO 3166-1 alpha-2 Country/Region code, AU: Australia; BR: Brazil; CA: Canada; CD: Democratic Republic of the Congo; CH: Switzerland; CN: China; FR: France; IL: Israel; MG: Madagascar; MN: Mongolia; MX: Mexico; PE: Peru; PH: Philippines; TH: Thailand; TW: Taiwan; US: United States of America; ZA: South Africa; ZM: Zambia. The outcome of each method is indicated by the following: *phy,* positive detection for phytoplasma; *oBa,* positive detection of another Bacteria; *neg*, negative detection; *phy-part* and *oBa-part* indicate partial *16Sr* sequence was obtained. Cq: quantification cycle value; **len**: lengths of sequences obtained by AHE (method 2) and Sanger sequencing; n. gen.: new genus; n. sp.: new species. Results of both AHE methods are available in Table S2.

Code	Tribe	Species	qPCR	Cq ^1^	AHE	len	nPCR-SS	len
1CH	Cicadulini	*Rhopalopyx elongata*	*phy*	**22.08**	*phy*	1530	*phy-part*	689
2TH	Athysanini	n. gen. T1 n. sp.	*phy*	**25.06**	*phy*	1529	*phy*	1318
3AU	Opsiini	*Orosius argentatus*	*phy*	**23.23**	*phy*	1528	*phy-part*	784
4CH	Athysanini	*Euscelidius variegatus*	*phy*	**26.41**	*phy*	1527	*phy*	1368
5MX	Scaphytopiini	*Scaphytopius aequus*	*phy*	**20.28**	*phy*	1527	*phy*	1378
6MG	Stenometopiini	Gen. sp.	*phy*	**27.20**	*phy*	1525	*phy-part*	783
7IL	Opsiini	*Neoaliturus argillaceus*	*phy*	**21.38**	*phy*	1525	*phy*	1366
8AU	Paralimnini	*Micrelloides* n. sp.	*phy*	**25.25**	*phy*	1524	*phy*	1317
9TW	Opsiini	*Alishania formosana*	*phy*	**25.85**	*phy*	1523	*phy*	1363
10US	Paralimnini	*Diplocolenus evansi*	*phy*	**20.57**	*phy*	1522	*phy*	1367
11FR	Fieberiellini	*Synophropsis lauri*	*phy*	**28.29**	*phy*	1522	*oBa-part*	812
12ZM	Selenocephalini	*Abimwa* sp.	*phy*	**27.65**	*phy*	1522	*oBa*	1386
13BR	Macrostelini	*Dalbulus maidis*	*phy*	**23.75**	*phy*	1397	*phy*	1381
14CN	Athysanini	*Nakaharanus bimaculatus*	*oBa*	31.21	*phy*	1522	*oBa-part*	795
15CA	Limotettigini	*Limotettix urnura*	*phy*	**29.26**	*phy*	1521	*oBa-part*	789
16FR	Chiasmini	*Doratura homophyla*	*phy*	**29.72**	*phy*	1527	*oBa-part*	784
17MN	Macrostelini	*Macrosteles guttatus*	*phy*	**26.21**	*phy*	1527	*phy*	1372
18MN	Paralimnini	*Adarrus* n. sp.	*oBa*	32.74	*phy-part*	1235 ^2^	*oBa*	1397
19CD	Scaphoideini	n. gen. ZA5 n. sp. 1	*oBa*	30.63	*phy-part*	1235 ^2^	*oBa*	1372
20PE	Chiasmini	*Exitianus obscurinervis*	*oBa*	31.82	*phy*	1518	*oBa*	1322
21ZA	Chiasmini	*Aconurella prolixa*	*oBa*	38.09	*phy*	1464	*oBa-part*	959
22CH	Scaphoideini	*Osbornellus auronitens*	*oBa*	31.27	*phy*	1527	*oBa*	1287
23CN	Scaphoideini	*Amimenus mojiensis*	*phy*	**27.80**	*phy*	1526	*phy*	1374
24FR	Scaphoideini	*Anoplotettix putoni*	*oBa*	32.73	*oBa*	1431	*oBa*	1224
25AU	Deltocephalini	n. gen. AU3 n. sp. 2	*oBa*	30.88	*phy-part*	910 ^2^	*oBa*	1394
26PH	Scaphoideini	n. gen. PH1 n. sp.	*phy*	**29.88**	*phy*	1527	*oBa*	1397
27US	Deltocephalini	*Graminella sonora*	*phy*	**21.13**	*phy*	1527	*phy*	1383
28ZA	Opsiini	*Neoaliturus angulatus*	*oBa*	30.99	*neg*	0	*oBa*	1288
29CN	Deltocephalini	*Paramesodes* sp.	*phy*	28.27	*phy-part*	620	*phy-part*	873
30MX	Deltocephalini	*Sanctanus fasciatus*	*oBa*	35.76	*oBa-part*	225	*oBa-part*	927
31PH	Scaphoideini	n. gen. PH1 n. sp. 2	*phy*	**27.83**	*neg*	0	*phy-part*	552
32BR	Scaphoideini	*Scaphoidula dentata*	*oBa*	33.05	*oBa-part*	621	*oBa-part*	1208
33US	Limotettigini	*Limotettix truncatus*	*oBa*	32.60	*neg*	0	*oBa*	1381
34AU	Deltocephalini	*Horouta aristarche*	*oBa*	34.79	*oBa-part*	399	*oBa-part*	1090
35BR	Deltocephalini	*Amplicephalus maculellus*	*phy*	**28.71**	*neg*	0	*oBa*	1277

^1^ In bold Cq value ≤ 30.38. ^2^ sequence not including the entire F2n/R2 fragment of *16Sr* gene.

**Table 2:** Contingency table of positive phytoplasma detection based on comparison of two traditional Polymerase Chain Reaction (PCR) methods, quantitative PCR (qPCR) and nested PCR followed by Sanger sequencing (nPCR-SS), and the new next generation sequencing method, Anchored Hybrid Enrichment (AHE). The outcome of the analyses is depicted by the following acronyms: *phy,* positive detection for phytoplasma; *oBa,* positive detection of another Bacteria; *neg*, negative detection. In gray results that are fully congruent among methods.

qPCR	nPCR-SS		AHE	
		*phy*	*oBa*	*neg*
*phy*	*phy*	15 (43%)	0	1 (3%)
*phy*	*oBa*	5 (14%)	0	1(3%)
*oBa*	*phy*	0	0	0
*oBa*	*oBa*	7 (20%)	4 (11%)	2 (6%)

**Table 3:** Identification and classification of the 23 phytoplasma strains detected in leafhoppers analyzed in this study. Newly designated subgroups are indicated in red. qPCR: quantitative PCR; nPCR-SS: nested PCR followed by Sanger sequencing; AHE: Anchored Hybrid Enrichment.

Code	Tribe	Species	16Sr Group Subgroup	Comparison
1CH	Cicadulini	*Rhopalopyx elongata*	16SrVI-L	qPCR = AHE = nPCR-SS
2TH	Athysanini	n. gen. T1 n. sp.	New group 1	qPCR = AHE = nPCR-SS
3AU	Opsiini	*Orosius argentatus*	16SrXXXVII-A	qPCR = AHE = nPCR-SS
4CH	Athysanini	*Euscelidius variegatus*	16SrI-F	qPCR = AHE = nPCR-SS
5MX	Scaphytopiini	*Scaphytopius aequus*	16SrI-B variant	qPCR = AHE = nPCR-SS
6MG	Stenometopiini	n. gen. n. sp.	New group 2	qPCR = AHE = nPCR-SS
7IL	Opsiini	*Neoaliturus argillaceus*	16SrIX-J	qPCR = AHE = nPCR-SS
8AU	Paralimnini	*Micrelloides* n. sp.	16SrXV-D	qPCR = AHE = nPCR-SS
9TW	Opsiini	*Alishania formosana*	New group 3	qPCR = AHE = nPCR-SS
10US	Paralimnini	*Diplocolenus evansi*	16SrXI-C	qPCR = AHE = nPCR-SS
11FR	Fieberiellini	*Synophropsis lauri*	16SrIII-U variant	qPCR = AHE
12ZM	Selenocephalini	*Abimwa* sp.	16SrIII-U variant	qPCR = AHE
13BR	Macrostelini	*Dalbulus maidis*	16SrI-B	qPCR = AHE = nPCR-SS
14CN	Athysanini	*Nakaharanus bimaculatus*	16SrIII-U variant	AHE
15CA	Limotettigini	*Limotettix urnura*	16SrXI-G	qPCR = AHE
16FR	Chiasmini	*Doratura homophyla*	16SrI-B	qPCR = AHE
17MN	Macrostelini	*Macrosteles guttatus*	16SrI-B	qPCR = AHE = nPCR-SS
20PE	Chiasmini	*Exitianus obscurinervis*	16SrIII-U variant	AHE
21ZA	Chiasmini	*Aconurella prolixa*	16SrIII-U variant	AHE
22CH	Scaphoideini	*Osbornellus auronitens*	16SrV-C	AHE
23CN	Scaphoideini	*Amimenus mojiensis*	16SrI-B	qPCR = AHE = nPCR-SS
26PH	Scaphoideini	n. gen. PH1 n. sp.	16SrI-AO	qPCR = AHE
27US	Deltocephalini	*Graminella sonora*	16SrI-B	qPCR = AHE = nPCR-SS

## Data Availability

Raw anchored hybrid reads were deposited in the Sequence Read Archive (SRA) of GenBank under BioProject PRJNA780295. SRA accession numbers are available in Table S2. Phytoplasma *16Sr* sequences obtained using AHE method 1, AHE method 2 and Sanger sequencing on the nested PCR template are also available in the Illinois Data Bank (DOI: https://doi.org/10.13012/B2IDB-9804959_V1 (accessed on 27 June 2022)) as fasta files. Other data presented in the study are included in the article: Tables S1–S3.

## Supplementary Materials

The following supporting information can be downloaded at: https://www.mdpi.com/article/10.3390/biology11070977/s1, Table S1: List of 35 samples of leafhoppers analyzed in this study. All taxa belong to subfamily Deltocephalinae (Hemiptera, Cicadellidae). Each sample represents one specimen of one species (columns B–E) collected in an independent collecting event (columns F–M); Table S2: Comparison of the sequences of phytoplasma *16Sr* assembled using Anchored Hybrid Enrichment (AHE) and predicted using 2 methods: ABySS (AHE_sequence_ABySS) and HybPiper (AHE_sequence_HybPiper). SRA_accession_number = SRA (Sequence Read Archive) accession number for raw reads; Table S3: Comparison between sequences of phytoplasma *16Sr* assembled using Anchored Hybrid Enrichment (AHE) and predicted using HybPiper method (used as Query) and sequences obtained using Sanger sequencing (used as Subject). Percentages of identity and coverage were obtained from BLASTN.
